# Remote Consultations for Monitoring Actinic Cheilitis: A Preliminary Non‐Randomized Clinical Trial

**DOI:** 10.1111/odi.70010

**Published:** 2025-06-19

**Authors:** Mônica Simões Israel, Nathália de Almeida Freire, Bruno Teixeira Gonçalves Rodrigues, Yasmin Muniz Dias, Manoela Domingues Martins, Carlos Augusto Moreira de Sousa, Vinicius Coelho Carrard

**Affiliations:** ^1^ Department of Diagnosis and Therapeutics, School of Dentistry Rio de Janeiro State University (UERJ) Rio de Janeiro Brazil; ^2^ Department of Oral Pathology, School of Dentistry Universidade Federal do Rio Grande do Sul (UFRGS) Porto Alegre Brazil; ^3^ Department of Information, Technologies and Health Education (DTIES), Faculty of Medical Sciences, Rio de Janeiro State University, Rio de Janeiro, Brazil Rio de Janeiro State University (UERJ) Rio de Janeiro Brazil; ^4^ TelessaudeRS‐UFRGS, Department of Epidemiology, School of Medicine Universidade Federal do Rio Grande do Sul (UFRGS) Porto Alegre Brazil; ^5^ Department of Oral Medicine, Otorhinolaringology Service Hospital de Clínicas de Porto Alegre (HCPA) Porto Alegre Brazil

**Keywords:** diagnosis, oral medicine, oral potentially malignant disorder, smartphone, telemedicine

## Abstract

**Objective:**

To evaluate the feasibility of remote consultation for monitoring actinic cheilitis.

**Materials and Methods:**

A cross‐over, non‐randomized clinical trial comparing remote and in‐person consultations for patients with actinic cheilitis. During the remote consultations, patients were interviewed and submitted clinical photos taken with smartphones. Local signs and symptoms were recorded, and examiners assessed whether a biopsy was indicated. Photos sent by patients were compared with those taken during the in‐person visits.

**Results:**

The study included 36 patients. The most frequent clinical presentation was plaques with erosive areas. Agreement between remote and in‐person evaluations regarding clinical alterations was 97% or higher. In 11% of cases (*n* = 4), both examiners recommended biopsy. In only 2 cases (5.6%), the remote examiner considered an in‐person consultation necessary. Photos taken by patients were rated as adequate in 88.6% of cases, compared to 97.1% for professional photos, with no statistically significant difference (*p* = 0.16).

**Conclusion:**

Teledentistry showed promising results for the follow‐up of actinic cheilitis and may help reduce healthcare costs. However, due to its potentially malignant nature, initial assessment must be performed by an oral medicine specialist to ensure appropriate diagnosis and management.

## 1 Introduction

Teledentistry (TD) is the remote delivery of dental care, guidance, education, and treatment using information technology, replacing the need for in‐person contact with patients. Telescreening and telemonitoring, components of TD, integrate information technology into routine dental practice, enabling remote triage and patient monitoring (Islam et al. 2022; Peng et al. 2020; Khan and Omar 2013). During the COVID‐19 pandemic, these resources gained visibility and became widely adopted to reduce unnecessary exposure for both patients and health professionals (Peng et al. 2020). Ultimately, evidence suggests that teledentistry is a promising clinical tool for preventing and promoting oral health, especially under the accelerated virtualization of dentistry (Fernández et al. 2021).

Several chronic conditions affect the oral cavity and require close monitoring, as there may be symptom exacerbation, drug resistance, or malignant evolution, such as with oral potentially malignant disorders (OPMD). Consequently, close follow‐up requires regular consultations, increasing costs for patients and the public health system. Telemonitoring can replace periodic in‐person visits with virtual consultations to monitor treatment outcomes and evaluate disease progression (Islam et al. 2022; Peng et al. 2020; Khan and Omar 2013; Mariño and Ghanim 2013).

Actinic cheilitis (AC) is an OPMD affecting predominantly the lower lip due to excessive and chronic exposure to ultraviolet B radiation from sunlight (de Santana Sarmento et al. 2014; Warnakulasuriya et al. 2021). AC commonly occurs in white‐skinned males over 45 years old, especially those working outdoors, such as sailors and farm workers. A cross‐sectional population‐based study observed that physical and chemical sun protection were associated with a lower occurrence of AC in individuals with greater exposure to the sun, thus emphasizing the importance of the orientation to patients regarding these factors (Lucena et al. 2022). Initially, vermilion atrophy accompanied by white plaques can be observed, progressing to squamous surfaces. These lesions, usually asymptomatic, appear white, red, or white with reddish areas (de Santana Sarmento et al. 2014; Carneiro et al. 2023). AC definitive diagnosis must be confirmed through an incisional biopsy and histopathological exam. Furthermore, histopathological grading of epithelial dysplasia remains the principal laboratory method for assessing the risk of malignant transformation in OPMD (Odell et al. 2021). Gradually, chronic ulcerations may develop, persisting for months and strongly suggesting progression to squamous cell carcinoma (SCC) (de Santana Sarmento et al. 2014; Carneiro et al. 2013; Rodrigues et al. 2020; Silva et al. 2020; Markopoulos et al. 2004; Dancyger et al. 2018).

This condition is relatively common, affecting a significant portion of the population in tropical countries (de Santana Sarmento et al. 2014). However, access to professionals trained in the assessment of changes that indicate a worsening of the disease, or even malignant transformation, is restricted (Brazilian Ministry of Health 2021). Therefore, there is a need to implement strategies to enable the assessment and follow‐up of AC patients. Based on this premise, the present pilot study evaluated whether monitoring patients with AC using teledentistry could enable virtual follow‐up and predict the best moment for an in‐person consultation.

## 2 Methods

### 2.1 Study Design and Ethical Consideration

This non‐randomized crossover clinical trial included subjects who were already patients of the Oral Medicine service of the State University of Rio de Janeiro (Rio de Janeiro, Brazil). Those patients had started AC monitoring through periodic consultations between June 2015 and April 2020. The local Research Ethics Council approved this study (CAAE protocol number: 64172622.0.0000.5347). This study was conducted in accordance with the CONSORT guidelines, ensuring transparency, completeness, and standardization in the reporting of randomized controlled trials.

### 2.2 Sample Size

In the present study, we have determined the sample size required to achieve a specificity of 0.99, to evaluate the biopsy necessity in online consultation versus in‐person consultation (gold standard), with a margin of error of 0.1, a significance level (*α*) of 0.05, and a statistical power of 0.80. The sample size calculation resulted in a required n of 36 cases. The sample size was obtained using the MKmisc r package.

### 2.3 Eligibility/Exclusion Criteria

All patients with clinical and histopathological criteria for AC seen at the Oral Medicine department of Rio de Janeiro State University with access to the internet and a smartphone were invited to take part in the study. No exclusion criteria were adopted.

### 2.4 Clinical Workflow

All AC patients requiring monitoring were invited to participate through telephone contact, featuring a census‐type sampling. Each patient was submitted to two types of consultation examinations: remote (online) and later in‐person (conventional). The two types of consultations were conducted by independent examiners. Both examiners underwent an initial training period that involved discussing assessment criteria using photographs of patients who were not included in the study. Subsequently, intra‐ and inter‐examiner calibration was conducted using photographs of 15 cases presented in digital format (via Google Forms). For each case, the examiners were required to determine whether the clinical features of interest were present or absent. After a 7‐day washout period, the evaluation was repeated. A minimum intraclass correlation coefficient (ICC) of 0.81 was observed, indicating strong agreement and demonstrating the reliability of the examiners. Both professionals were oral medicine specialists with at least 10 years of clinical experience.

### 2.5 Remote (Online) Consultation

Before the online interview, the patient was instructed to take three standardized photographs of the vermillion and lower lips for evaluation of the quality of the acquired data. An illustrated digital leaflet was developed to guide patients in taking photographs remotely using a smartphone. Patients were advised to enlist the help of another person to capture the images. The first step involved the patient sitting on a chair with a backrest in a naturally lit area without direct sunlight. The first photograph was taken with the camera positioned perpendicular to the face, with the patient's lips relaxed. The second image required the mouth to be slightly open, with the camera tilted both upward and downward. Finally, the third photograph was taken with the mouth fully open and the camera once again in a perpendicular position. The photographs taken by the patients were sent through the online platform (WhatsApp LLC, Menlo Park, CA) to the examiner, who determined whether the photographs were suitable for evaluation. If necessary (poor focus and/or brightness), the patient was instructed to retake the photographs.

Once the photos were analyzed, the consultation began. First, an interview was conducted using a Google Form, which was filled out by the examiner. The presence of symptoms (pain, itching, dryness, and bleeding) and the use of photoprotection were objectively questioned. By comparing the reported exposure pattern with those noted in previous consultations, the examiner recorded the current status (no exposure, reduced exposure, or continued exposure to UV radiation).

After that, the calibrated examiner (examiner 1) analyzed the photographs, looking for the following signs: desquamative areas, pigmented sites, plaques, erosions, or ulcers. Additionally, an assessment of the case's evolution (improvement, worsening, or maintenance of previously observed characteristics) was made by comparing the photo sent via WhatsApp with the one retrieved from the service's database, as photographic records are routinely made at each consultation. Based on the data collected during the remote consultation, the examiner had to determine if the case required a new incisional biopsy because of the presence of suspicious signs. The criteria used to support that decision‐making were the presence of new plaque(s), erosion(s), or ulcer(s).

### 2.6 In‐Person (Conventional) Consultation

Immediately after the remote consultation, the patients went to the outpatient clinic for a conventional consultation. At this point, the second evaluator (examiner 2) carried out the conventional monitoring consultation, which followed the same criteria considered in the remote consultation. Photos were taken with a digital single‐lens reflex (SLR) camera following the standardized positions in the leaflet provided to the patient. At the end of this consultation, based on the aforementioned clinical criteria, the examiner defined whether a biopsy was necessary (or not).

### 2.7 Statistical Analysis

The data were collected and organized using REDCap software and analyzed using R statistical software. The population was characterized by relative and absolute frequencies for categorical variables, and by mean and standard deviation for numerical variables.

Sensitivity, specificity, and accuracy measures were calculated to compare the results of the teledentistry consultation with the gold‐standard reference, which was the in‐person dental consultation in the same population. A ROC curve was also constructed to compare the outcomes between the consultations. Agreement between the consultations was assessed using the Kappa index.

## 3 Results

Thirty‐six patients were included in the study. The sample was predominantly male (55.6%, *n* = 20). The mean age of the patients was 60 years, ranging from 38 to 86 years. Regarding symptoms, most patients were asymptomatic (*n* = 24, 66.7%), but 30.6% (*n* = 11) reported dryness. Interestingly, approximately 38% of the individuals evaluated did not use lip balm, even after being advised to do so. Further details about sample characteristics are provided in Table 1.

**TABLE 1:** Epidemiological and clinical profile of the sample (*n* = 36).

Variable	Values
Age (years)	
Mean (SD)	60.1 (10.4)
Min–max	38–86
Sex, *n* (%)	
Male	20 (55.6)
Female	16 (44.4)
Symptoms, *n* (%)	
No	24 (66.7)
Yes	12 (33.3)
Pain, *n* (%)	
No	34 (94.4)
Yes	2 (5.6)
Dryness, *n* (%)	
No	25 (69.4)
Yes	11 (30.6)
Itching, *n* (%)	
No	34 (94.4)
Yes	2 (5.6)
Bleeding, *n* (%)	
No	34 (94.4)
Yes	2 (5.6)
Lip balm use	
Never	14 (38.9)
Rarely	11 (30.6)
Twice a day (or more)	11 (30.6)
Lip balm (level of photoprotection)	
Do not use	13 (36.1)
< 30 SPF	3 (8.3)
30 SPF	5 (13.9)
> 30 SPF	12 (33.3)
Unknown	3 (8.3)
Cap/Hat use	
No	16 (44.4)
Yes	20 (55.6)

Abbreviation: SFP, sun protection factor.

**FIGURE 1:**
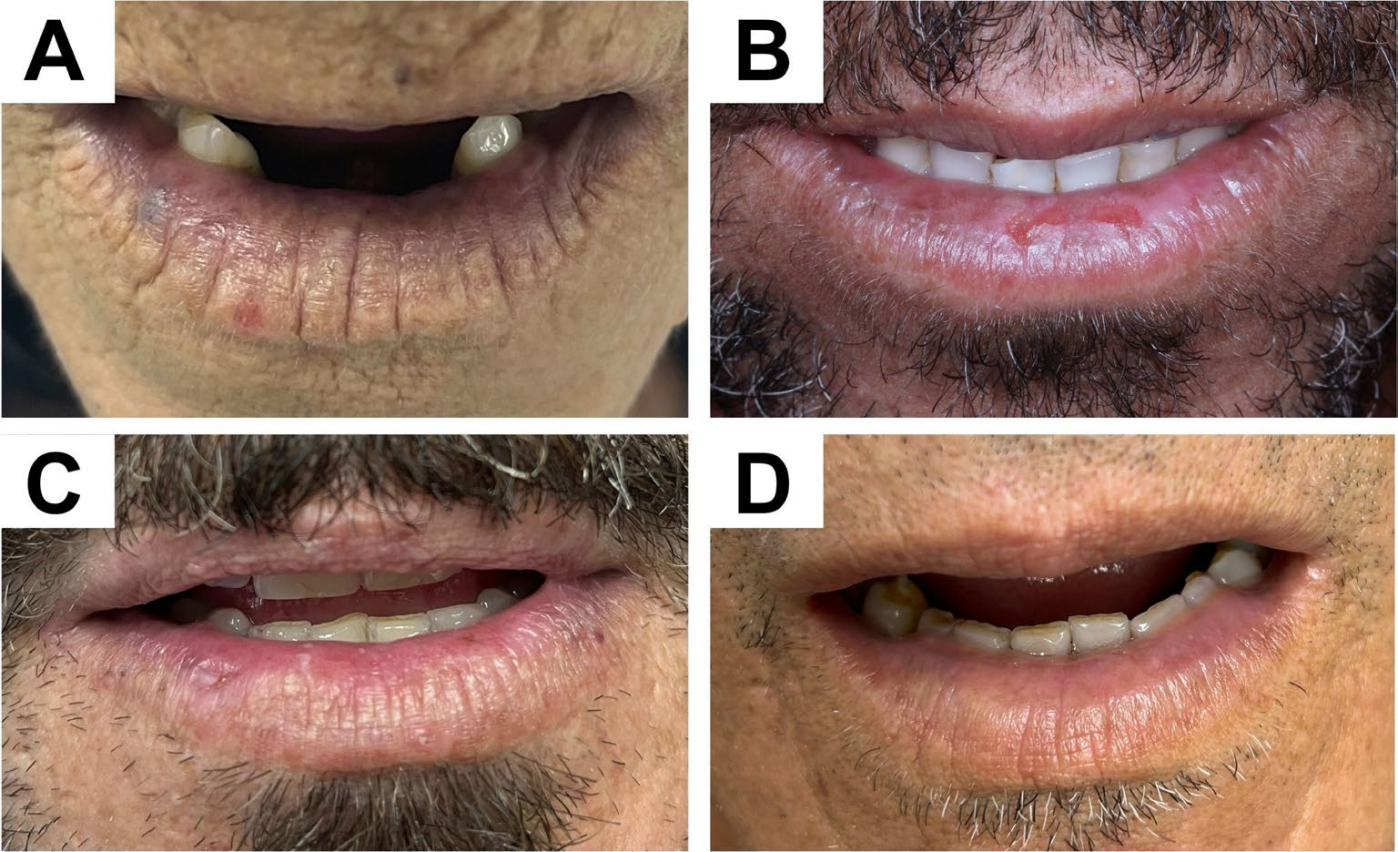
Representative images of some evaluated patients. (A) Diffuse white plaque, accompanied by a venous lake on the right side of the lower lip. (B) Painful ulceration associated with whitish plaques. (C, D) Patients presenting whitish plaques associated with erosive areas without symptoms.

Regarding the clinical signs observed between the online and in‐person consultations, there was a 100% concordance on the following items: desquamation and ulcers. However, a disagreement was found in only one patient—at the remote consultation, pigmentation and erosion were presented, while none of these findings could be encountered at in‐person consultation, but the presence of plaques. Table 2 summarizes the percentage agreement between consultations. Considering that face‐to‐face consultation remains the gold standard for clinical evaluation, and acknowledging that photographic assessment may be subject to some degree of discrepancy, no clinical intervention was undertaken in this case. Representative photos of the evaluated cases may be observed in Figure 1.

**TABLE 2:** Comparison between both types of consultation.

Online consultation, *n* (%)	In‐person consultation, *n* (%)		
		Desquamation	
		Yes	No
Desquamation	Yes	11 (100.0)	0 (0.0)
	No	0 (0.0)	25 (100.0)
		Plaque	
		Yes	No
Plaque	Yes	26 (100.0)	1 (10.0)
	No	0 (0.0)	9 (90.0)
		Pigmentation	
		Yes	No
Pigmentation	Yes	10 (100.0)	1 (3.8)
	No	0 (0.0)	25 (96.2)
		Erosion	
		Yes	No
Erosion	Yes	4 (100.0)	1 (3.1)
	No	0 (0.0)	31 (96.9)
		Ulcer	
		Yes	No
Ulcer	Yes	4 (100.0)	0 (0.0)
	No	0 (0.0)	32 (100.0)
		Biopsy	
		Yes	No
Biopsy*	Yes	5 (100.0)	0 (0.0)
	No	0 (0.0)	31 (100.0)

* Biopsy specificity (IC 95%): 1.00 (0.89, 1.00).

The follow‐up consultation data revealed various outcomes regarding lip photoprotection, clinical evolution, and further recommendations. Regarding the adoption of lip photoprotection, 19.4% of patients did not use any photoprotection, while 41.7% reduced UV radiation exposure, and 38.9% maintained exposure. In terms of clinical evolution, 5.6% of cases showed worsening, 55.6% reported no additional clinical alterations, and 38.9% experienced improvement. Recommendations for in‐person consultations were provided for 19.4% of cases, whereas 77.8% were not advised to attend in‐person consultations, and 2.8% were deemed not applicable. As for biopsy recommendations, 13.9% of cases required a biopsy, while 86.1% did not receive this recommendation.

**FIGURE 2:**
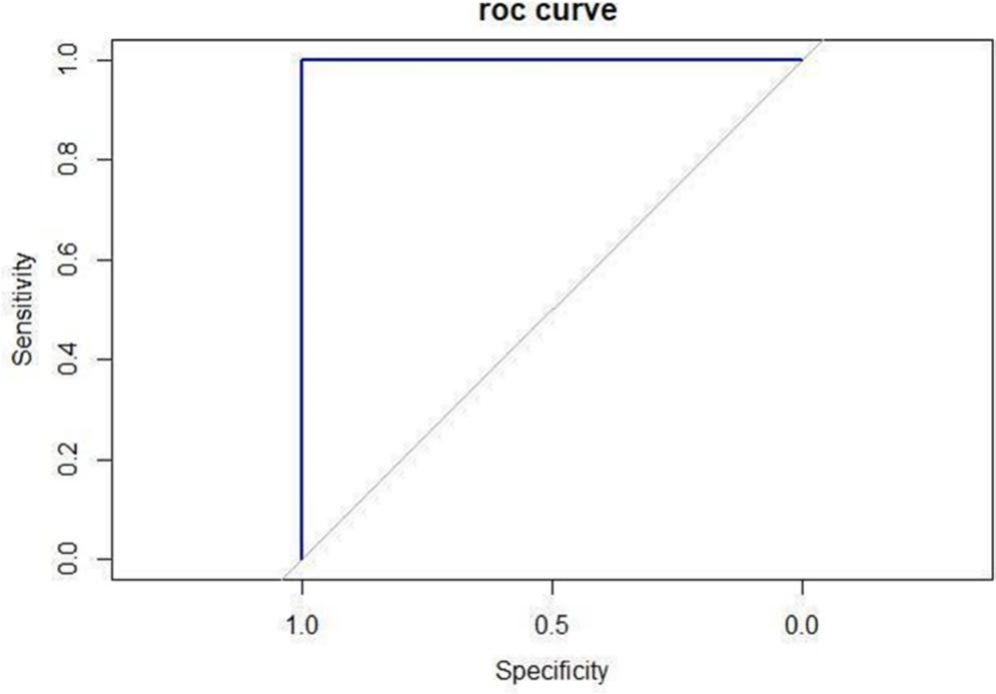
ROC curve of the accuracy between the clinical signs identification of in‐person versus remote consultation.

Table 3 summarizes the accuracy, sensitivity, specificity, and Kappa coefficient values from the comparison between clinical signs identified during in‐person and remote consultations. Data from the remote and in‐person consultations showed 100% agreement regarding the need for a new incisional biopsy in five cases (13.9%). The remote evaluation performance, according to the ROC curve, was considered excellent (AUC = 1). The proximity of the curve to the Y‐axis indicates high sensitivity and specificity at different cut‐off points (Figure 2).

**TABLE 3:** Accuracy, sensitivity, specificity, and kappa coefficient values for comparisons between the clinical signs' identification of in‐person versus remote consultation.

Clinical sign	Accuracy	Sensitivity	Specificity	Agreement (Kappa coefficient)
Desquamation	1.00	1.00	1.00	1.00
Plaque	0.97	1.00	0.90	0.93
Pigmentation	0.97	1.00	0.96	0.93
Erosion	0.97	1.00	0.97	0.87
Ulcer	1.00	1.00	1.00	1.00

## 4 Discussion

AC is a common OPMD affecting the lips that presents a risk for malignant transformation. Monitoring AC patients typically involves regular in‐person consultations, which can be burdensome for both patients and healthcare systems, especially in regions with limited access to specialists (Carneiro et al. 2023; Petruzzi and De Benedittis 2016; Martins Filho et al. 2011; Rodríguez‐Blanco et al. 2018; Moreira et al. 2021). This is the first study assessing the feasibility of remotely monitoring patients with AC. The main results showed a high degree of agreement between the two types of evaluation, indicating that the strategy is promising. Additionally, it was demonstrated that the photos taken by patients were of sufficient quality to be analyzed by specialists, who felt confident in monitoring without direct contact with the patients.

The satisfactory performance of remote evaluation compared to in‐person consultation was expected, since two previous studies with oral mucosal lesions had shown similar results (Fonseca et al. 2022; Flores et al. 2022). However, the present study emphasizes the possibility of using this tool for a specific condition, even when specific clinical signs are independently evaluated. These results support the use of this strategy in certain population groups in which the condition is prevalent, such as rural workers. Considering these groups do not have easy access to or regular opportunities to travel to health services, this approach emerges as an alternative with remarkable potential. Adopting the approach tested in this study, the need for in‐person consultations would be reserved only for situations where the most important clinical signs are identified. In the context of this study, this group represented approximately 20%, which would significantly reduce the demand for centers where specialists are available.

The systematic review conducted by Flores et al. (2020) observed that teledentistry has the potential to improve the quality of care related to the diagnosis and management of oral lesions, by shortening the distance between patients who require specialized diagnoses and specialists. In our study, 97% of remote evaluators reported feeling confident in their assessments, despite not having direct contact with patients. This finding aligns with previous literature suggesting high satisfaction and confidence levels among healthcare providers conducting remote consultations (Carrard et al. 2018; Higgins et al. 2017; Roxo‐Gonçalves et al. 2023; Smith et al. 2019). Moreover, there was a high level of agreement between remote and in‐person assessments, including the decision to proceed with a biopsy based on clinical suspicion. The only case of disagreement between the in‐person versus remote consultation could be explained by the limited quality of photos sent by the patient, compromising a more accurate evaluation. Therefore, low‐quality pictures should initially be filtered and not accepted for evaluation, requiring the patient to retake them according to the standardized photographs. The ROC curve analysis also corroborates the satisfactory performance of that alternative approach.

However, it is essential to acknowledge the limitations of our study. The relatively small sample size and the subjective nature of the assessment criteria hinder the generalizability of our findings (Greenhalgh et al. 2018). On the other hand, it is important to note that clinical trials addressing AC typically involve small samples (Bezerra et al. 2019; Rosenthal et al. 2019; Andreadis et al. 2020; Arisi et al. 2022). Additionally, factors such as lighting conditions and patient cooperation may have influenced the quality of the remotely captured images, potentially affecting diagnostic accuracy (Lee et al. 2014; Gangwani et al. 2024). Furthermore, given Brazil's vast territorial extension and pronounced socioeconomic disparities, limited access to high‐speed internet and mobile devices can pose significant barriers to the effective implementation of teledentistry. The elderly population, in particular, may experience difficulties navigating digital tools, and challenges related to digital literacy suggest that this approach may not be suitable for all patients.

Nevertheless, it is important to emphasize that the region where our team operates has had a telediagnosis service in place for more than 5 years (Carrard et al. 2018; Roxo‐Gonçalves et al. 2023). This infrastructure enables timely identification of suspicious lesions and facilitates prompt referral for in‐person consultation with a specialist whenever necessary, thereby strengthening the integration between primary care and specialized services.

Although promising, the results obtained so far are preliminary. Confirmatory studies with larger sample sizes are warranted to validate our findings before adopting this approach as a routine (Bhaumik et al. 2020; Prensky 2012). If future research confirms the efficacy of remote assessment, it could revolutionize healthcare delivery, especially in regions with limited access to stomatologists. By reducing the need for patient travel and alleviating long wait times for conventional consultations, this approach could significantly improve access to and quality of oral healthcare services.

## 5 Conclusion

Our findings suggest that teledentistry may serve as a complementary tool for monitoring AC, particularly in cases presenting with mild clinical features. The high agreement between remote and in‐person consultations regarding clinical signs and biopsy recommendations supports the feasibility of this approach. However, remote monitoring cannot replace in‐person specialist evaluation, especially in cases of clinical progression or atypical presentations. Given the high prevalence of AC in Brazil and its relatively low malignant transformation potential compared to other oral potentially malignant disorders (OPMDs), a stratified monitoring strategy may be appropriate. This approach—combining remote follow‐up for milder cases with prompt referral for specialist assessment when needed—could help optimize resource allocation, particularly in public healthcare systems with limited access to oral medicine specialists.

Furthermore, as AC predominantly affects older individuals with low health literacy, patient education and awareness initiatives remain crucial to ensure timely diagnosis and treatment. It is also important to recognize that this approach may not be feasible for all individuals in Brazil, due to economic limitations that restrict access to mobile phones, reliable internet connections, and digital literacy. These barriers must be considered when designing and implementing remote monitoring strategies to ensure equitable access to care.

## Author Contributions

**Mônica Simões Israel:** conceptualization, writing – original draft, investigation, methodology, validation, writing – review and editing, project administration, supervision. **Vinicius Coelho Carrard:** investigation, writing – original draft, conceptualization, methodology, validation, visualization, writing – review and editing, formal analysis, project administration, supervision. **Carlos Augusto Moreira de Sousa:** methodology, formal analysis, project administration, supervision, writing – review and editing, writing – original draft, software. **Bruno Teixeira Gonçalves Rodrigues:** writing – original draft, writing – review and editing, investigation, methodology. **Nathália de Almeida Freire:** writing – original draft, writing – review and editing, investigation, methodology, visualization. **Manoela Domingues Martins:** supervision, writing – original draft. **Yasmin Muniz Dias:** writing – original draft, writing – review and editing, visualization.

## Conflicts of Interest

The authors declare no conflicts of interest.

## Data Availability

The data that support the findings of this study are available on request from the corresponding author. The data are not publicly available due to privacy or ethical restrictions.
